# Contaminants in commercial preparations of ‘purified’ small leucine-rich proteoglycans may distort mechanistic studies

**DOI:** 10.1042/BSR20160465

**Published:** 2017-01-13

**Authors:** Sharon J. Brown, Heidi R. Fuller, Philip Jones, Bruce Caterson, Sally L. Shirran, Catherine H. Botting, Sally Roberts

**Affiliations:** 1 Robert Jones & Agnes Hunt (RJAH) Orthopaedic Hospital NHS Foundation Trust & ISTM (Keele University), Oswestry, SY10 7AG, U.K.; 2 Cardiff School of Biosciences, The Sir Martin Evans Building, Museum Avenue, Cardiff, CF10 3AX, U.K.; 3 BSRC Mass Spectrometry and Proteomics Facility, Biomedical Sciences Research Complex, University of St Andrews, North Haugh, St Andrews, Fife, KY16 9ST, U.K.

**Keywords:** biglycan, commercial preparations, decorin, glycosaminoglycan, mass spectrometry, small leucine-rich proteoglycans (SLRPs)

## Abstract

The present study reports the perplexing results that came about because of seriously impure commercially available reagents. Commercial reagents and chemicals are routinely ordered by scientists and expected to have been rigorously assessed for their purity. Unfortunately, we found this assumption to be risky. Extensive work was carried out within our laboratory using commercially sourced preparations of the small leucine-rich proteoglycans (SLRPs), decorin and biglycan, to investigate their influence on nerve cell growth. Unusual results compelled us to analyse the composition and purity of both preparations of these proteoglycans (PGs) using both mass spectrometry (MS) and Western blotting, with and without various enzymatic deglycosylations. Commercial ‘decorin’ and ‘biglycan’ were found to contain a mixture of PGs including not only both decorin and biglycan but also fibromodulin and aggrecan. The unexpected effects of ‘decorin’ and ‘biglycan’ on nerve cell growth could be explained by these impurities. Decorin and biglycan contain either chondroitin or dermatan sulfate glycosaminoglycan (GAG) chains whereas fibromodulin only contains keratan sulfate and the large (>2500 kDa), highly glycosylated aggrecan contains both keratan and chondroitin sulfate. The different structure, molecular weight and composition of these impurities significantly affected our work and any conclusions that could be made. These findings beg the question as to whether scientists need to verify the purity of each commercially obtained reagent used in their experiments. The implications of these findings are vast, since the effects of these impurities may already have led to inaccurate conclusions and reports in the literature with concomitant loss of researchers’ funds and time.

## Introduction

For several decades, it has been known that extracellular matrix (ECM) molecules can provide topographical cues and guidance for neuronal growth. Proteoglycans (PGs) are a major component of the ECM in several tissues in our body and have been shown to influence nerve growth in many systems from the developing central nervous system [1,2] to the intervertebral disc [3–5]. For example, they are reported to inhibit outgrowth of sensory neurons [6], reduce astrogliosis or, indirectly, promote axon growth of adult sensory neurons [7], with chondroitin sulfate–PGs acting through receptor protein tyrosine phosphatase σ [8]. PGs are diverse molecules that can range in molecular weight from 25 kDa to 3 million kDa, depending on the length of their protein core and the number and type of glycosaminoglycan (GAG) chains attached (which can vary from 1 to 150). In addition, PGs may occur as a single molecule or, as in the case of aggrecan or versican, be linked to hyaluronan to form very large aggregates. The exact attribute of the PG that affects nerve growth is not clear as it may be the core protein, GAG side chains, GAG sulfation, the degree of aggregation in the case of aggrecan or a combination of these factors.

One of the main areas of interest within our research group is the biology and degeneration and regeneration of the intervertebral disc. Degeneration of the disc is a common phenomenon, which is of interest clinically because of its association with one of the largest health burdens in the Western world, that of back pain [9–12]. Previously, a large twin study found degeneration of the disc in the lumbar spine to be the single most important risk factor for low back pains [13]. The intervertebral disc is a connective tissue that is unusual in having little vascularization or innervation. In the healthy disc, nerves are restricted to the outer few millimetres of the disc; in the lumbar region, responsible for most pathology, this is typically between 4 and 5 mm. When discs become degenerate, the effects can be widespread resulting in abnormal loads being transmitted to the adjacent tissues and structures [14] and are typified by microscopic and biochemical changes, with altered cellular metabolism and structural matrix derangements [15]. The loss of PG and water from the matrix leads to cracks and fissures forming within the disc and its adjacent endplates, and these are often accompanied by neural and vascular ingrowth [16–19].

PGs, with their considerable hydrophilic capacity, are fundamental to the main function of the disc, that of weight bearing in the spine and it is these molecules that are the first to alter when discs degenerate [20]. Aggrecan is the main PG in the disc and it becomes enzymatically fragmented during age-related degeneration, both in terms of the protein core being cleaved and the GAG chains becoming shorter, resulting in the reduced PG and GAG content in degenerative discs [21]. In previous studies, our group have demonstrated that disc aggrecan inhibits the growth of neurites in a dose dependent manner [3], hence providing a mechanism and linkage for the loss of PG and increased innervation in degenerate discs.

Although PGs make up 13–61% of the dry weight in the disc [22] and the majority of this is aggrecan, there are other PG family members present in the matrix, albeit at a lower concentration, such as the small leucine-rich proteoglycans (SLRPs). Several SLRPs are known to occur in human discs and include biglycan, decorin, fibromodulin, keratocan and lumican [23–26]. In addition, some of these SLRPs have been shown to be increasingly fragmented with disc degeneration in humans [23], as well as in the discs of chondrodystrophoid breeds of dog, which are prone to disc disorders [27]. In addition to aggrecan, decorin and biglycan in particular have been shown to influence nerve growth and function. For example, decorin promotes both axon growth in spinal cord injury in the rat [7] and influences scar formation at the site of nerve injury [28] which in turn affects nerve growth whereas biglycan contributes to synapse stability [29]. Decorin together with TGFβ (which binds to decorin and modulates its affect) may have a biphasic expression in nerve injury, likely to be part of a nerve repair mechanism [30].

The present study highlights the importance of evaluating the composition of commercially available preparations. We had set out to determine if decorin and biglycan might influence nerve growth in model systems used previously in our laboratory to study the influence of aggrecan [3,31,32], but unexpected results led us to investigate the composition of both commercially purchased SLRP molecules. This in turn has led us to be increasingly sceptical about the purity of some commercially available biochemicals.

## Materials and methods

### Culture and assessment of neuronal growth

Dorsal root ganglia (DRGs) were obtained as previously described and used as a source of sensory neurons [3]. Briefly, DRGs were dissected from white leghorn chickens at day 10 of embryogenesis, cut into quarters and then at least five explants were seeded on to a Petri dish coated with various substrates. These included the neural-permissive type I collagen and either ‘decorin’ (D8428, Sigma–Aldrich, lot numbers 101M4033V and 029k4058) or ‘biglycan’ (B8041, Sigma–Aldrich, lot number 070M4068). The substrate coatings were prepared by flooding the wells of a 12-well tissue culture plate with a solution of nitrocellulose in methanol (0.4 cm^2^/ml) that was then allowed to dry. Strips of Whatman filter paper, soaked in solutions of ‘decorin’ ranging from 10 to 1000 µg/ml or ‘biglycan’ ranging from 2 to 500 µg/ml diluted in PBS with 10% rhodamine-B, were blotted on to the nitrocellulose and allowed to dry. The filter paper was removed and the remainder of the wells were coated with type I collagen from rat tail (Collaborative Biomedical) at a concentration of 100 µg/ml in PBS. The DRG-seeded Petri dishes were maintained in explant culture medium (Dulbecco’s modified Eagle’s medium (DMEM)/Ham’s F-12 supplemented with progesterone, insulin, sodium selenite, phosphocreatine, transferrin and sodium pyruvate) containing 15 ng/ml nerve growth factor (βNGF; R&D Systems) at 39°C for 72 h prior to fixation in 4% paraformaldehyde. Neurite growth was monitored by phase-contrast and fluorescent microscopy and measured on captured images as described previously [3,31]. A minimum of 20 DRG explants for each experimental condition were assessed and scored.

### Proteoglycans for nerve culture and Western blot assays

Both ‘decorin’ and ‘biglycan’ were obtained from Sigma–Aldrich and had been prepared from bovine articular cartilage. Certificate analyses of these products stated that protein levels had been analysed [33] in the lyophilized preparations and they complied with their specifications, which for ‘decorin’ was to contain ≥45% protein and for ‘biglycan’ to contain ≥30% protein. In addition it reported that GAG content, as determined by dimethylmethylene blue (DMB), was a minimum of 55% GAG for the ‘decorin’ and 70% for the ‘biglycan’ with uronic acid (as detected by carbazole) comprising between 20 and 27% of this GAG content for the ‘decorin’ and 14% for ‘biglycan’. Following enzymatic treatment with chondroitinase ABC, the major band reported on the Certificates of Analysis for both ‘decorin’ and ‘biglycan’ was reported to be a 45 kDa protein band following separation on 10-15% SDS-PAGE electrophoresis gel, as detected by Coomassie Blue staining.

To investigate whether the GAG chains present on the SLRPs inhibit nerve growth, different enzymes were used to selectively remove specific GAG chains from ‘decorin’ and ‘biglycan’. Chondroitinase AC (Seikagaku Corporation, Japan), which was used at concentrations ranging from 2 to 250 mU*/*ml, cleaves unsulfated chondroitin, chondroitin-4-sulfate and chondroitin-6-sulfate from the core protein and chondroitinase ABC (also used over a range of 2–250 mU/ml; Sigma–Aldrich) cleaves all of these in addition to dermatan sulfate (DS). Filter paper strips previously blotted with 500 µg/ml ‘decorin’ or ‘biglycan’ were incubated with the enzymes at 37°C for 2 h.

Aggrecan was prepared using previously described methods [21,34] and was extracted from the nucleus pulposus of a disc obtained postmortem from a 70-year-old male. Briefly, as described by Sivan et al. [35], the disc was diced and aggrecan was extracted using ten volumes of 4 M guanidine hydrochloride (GuHCl) containing proteinase inhibitors in 50 mM Tris buffer, pH 7.4 at 4°C for 48 h [36]. Using centrifugation (10000 rpm for 40 min at 4°C), the supernatant was dialysed in the same buffer but without GuHCl. This fraction contained both aggrecan and its degradation products; using direct dissociative CsCl density gradient centrifugation in 4 M GuHCl (50 mM Tris buffer, pH 7.4) starting at a density of 1.5 g/ml (10°C, 50000 rpm for 48 h), the aggrecan monomers were separated in the highest density gradient fraction according to their buoyant density and molecular weight [37]. The purified human aggrecan was dialysed against distilled water and freeze-dried.

### Western blot analysis of ‘decorin’, ‘biglycan’ and aggrecan

Western blotting was carried out as previously described [38] and repeated a minimum of three times. A pre-stained molecular weight standard (SeeBlue-2, Invitrogen) was run together with extracts of bovine nasal cartilage, and SLRPs and aggrecan isolated from human intervertebral disc, as positive controls, which produced bands in the region of expected molecular weight (results not shown).

PGs were deglycosylated prior to Western blotting. To remove chondroitin sulfate (CS) and DS chains, the commercially sourced ‘decorin’ and ‘biglycan’ and purified human aggrecan were digested with chondroitinase ABC or chondroitinase AC (both at 250 mU/ml in Tris acetate buffer) or left undigested for 2 h at 37°C. In addition, they were digested with chondroitinase ABC (200 mU/ml) and keratanase (100 mU/ml) and keratanase II (2 mU/ml) in Tris acetate buffer to ensure removal of any CS, DS and keratan sulfate (KS) GAG chains. The enzymatically digested or undigested PGs were mixed with lithium dodecyl sulfate (LDS) PAGE application buffer (4×) and 500 mM dithiothreitol (10×) then heated for 30 min at 70°C, cooled and either 10 µg or 25 µg were loaded into each well and separated on 4–12% Bis-Tris gradient gels for 35 min at a constant voltage of 200 V (Novex, Invitrogen) under reducing conditions.

Two protocols for transferring to the nitrocellulose membranes and visualization were used. For **Protocol 1**, the separated PGs (which had been digested with chondroitinase AC or ABC) were transferred to nitrocellulose membranes (0.22 µm) using NuPAGE transfer buffer plus 10% methanol at a constant voltage of 30 V for 1 h. The membranes were blocked for 1 h with 5% skimmed milk/0.1% Tween 20 in 50 mM Tris-HCl and 0.15 M NaCl, pH 7.2 (TBS). Primary mouse monoclonal antibodies against GAGs (5D4, 2B6 and 3B3) at the concentrations provided in Table 1 were then applied to the membranes for 1 h. Membranes were rinsed in TBS and a secondary antibody applied (Mouse IgG Vectastain ABC Kit, Vector Laboratories) for 30 min followed by the avidin/biotin complex for an additional 30 min. Following colour development with 3,3΄-diaminobenzidine (DAB), membranes were rinsed in distilled water and allowed to dry.

**Table 1:** Summary of the antibodies used during Western blotting to identify the presence of decorin, biglycan, aggrecan, fibromodulin and lumican core proteins and KS glycosaminoglycan chains and CS neo-epitopes (‘stubs’) following enzymatic treatment

Antibody	Dilution	Detects	Epitope	Reference
mAb DS-1	1:125	Decorin	C-terminal of core protein	[44]
pAb PR-85	1:500	Biglycan	C-terminus (CGG)TDRLAIQFGNYKK	[45]
mAb 6B4	1:50	Aggrecan	Amino acids 413–424 of human aggrecan	[46]
pAb PR-184	1:500	Fibromodulin	C-terminus (CGG)LRLASLIEI	[45]
pAb PR-586	1:500	Lumican	C-terminus (CGG)LRVANEITVN	[47]
mAb 5D4	1:500	KS	Over-sulfated KS heptasaccharides	[48]
mAb 1B4	1:2500	KS	Low-sulfated KS	[49]
mAb 2B6	1:50	C-4-S ‘stubs’	4-sulfated unsaturated disaccharide CS neo-epitopes	[50]
mAb 3B3	1:50	C-6-S ‘stubs’	6-sulfated unsaturated disaccharides CS neo-epitopes	[51]

All antibodies were provided by B. Caterson (Cardiff University) except for those against biglycan, fibromodulin and lumican that were kindly provided by Peter Roughley (McGill University), and to decorin (purchased from Developmental Studies Hybridoma Bank, Iowa).

In **Protocol 2**, following separation via electrophoresis as described above, the commercial SLRPs (which had been digested with chondroitinase ABC, keratanase and keratanase II) were transferred to nitrocellulose membranes using an iBlot system (Program 3) and associated transfer stacks (Invitrogen). These membranes were then probed for decorin, biglycan, aggrecan, fibromodulin, lumican and KS using the previously described primary antibodies (Table 1). The primary antibodies were applied using iBlot detection stacks and appropriate chromogenic kits (Invitrogen) followed by the appropriate secondary antibody (anti-rabbit at 1:2000 or anti-mouse at 1:5000). Membranes were rinsed in wash solution (Invitrogen) followed by autoclaved distilled water and the BCIP/NBT chromogenic substrate applied and colour development allowed for a maximum of 1 h.

### Protein identification by MS

Two different batches of the commercially sourced ‘decorin’ and one batch of ‘biglycan’ were enzymatically digested for removal of CS, DS and KS as described for Western blotting, before being separated on 12.5% SDS-PAGE gel for MS. Bovine nasal cartilage was treated similarly and used as a positive control. Following Coomassie Blue staining of the 12.5% gel, protein slices, spanning the entirety of each lane were excised and digested overnight at 37°C with trypsin (porcine, sequencing grade, Promega), according to the method described by Shevchenko et al. [39]. Briefly, gel slices were washed with 0.1 M NH_4_HCO_3_/acetonitrile (1:1), reduced with 10 mM dithiothreitol in 0.1 M NH_4_HCO_3_ at 56°C for 45 min followed by alkylation of cysteine residues by incubation with 55 mM iodoacetamide in 0.1 M NH_4_HCO_3_ for 30 min at room temperature. Prior to MS analysis, the protein digests were cleaned up and concentrated using C18 Ziptips according to the manufacturer’s recommendations (Millipore). Peptides were eluted from the Ziptips on to a stainless steel MALDI target plate with α-cyano-4-hydroxycinnamic acid (CHCA) matrix at 3 mg/ml (in 70% acetonitrile and 0.1% 2,2,2-trifluoroacetic acid (TFA)) and allowed to dry. Close external peptide standards were used to calibrate the instrument in both MS and MS/MS mode. Both MS and MS/MS analysis of the peptides was performed using a 4800 MALDI TOF/TOF (AB Sciex). The mass spectrometer was operated under the control of 4000 Series Explorer software (V3.5.2 Applied Biosystems). Peak lists of MS and MS/MS spectra were also generated with the 4000 Series Explorer software and the following parameters were used after selective labelling of monoisotopic mass peaks: MS peak lists: S/N threshold 3, Savitzky–Golay smoothing with 3 points across peak (FWHM), no baseline correction, MS/MS peak lists: S/N threshold 10, Savitzky–Golay with 7 points across peak (FWHM) and no baseline correction. Automated database searches were run using GPS Explorer software (V3.6, AB Sciex). Using MASCOT as the search engine, the Swissprot database was explored using the following parameters: precursor ion mass tolerance of 0.15 Da, fragment ion mass tolerance of 0.15 Da, mammalian taxonomy and finally, carbamidomethyl modification of cysteines and oxidation of methionine residues were allowed as variable modifications.

### Statistical analysis

The non-parametric statistical test, Kruskal–Wallis with post-hoc analysis (Dunn’s multiple comparisons test), was used to determine any differences in neurite growth on different substrata. Statistical significance was considered at *P*<0.05.

## Results

### Effect of ‘decorin’ and ‘biglycan’ on DRG neurite growth

**Figure 1:**
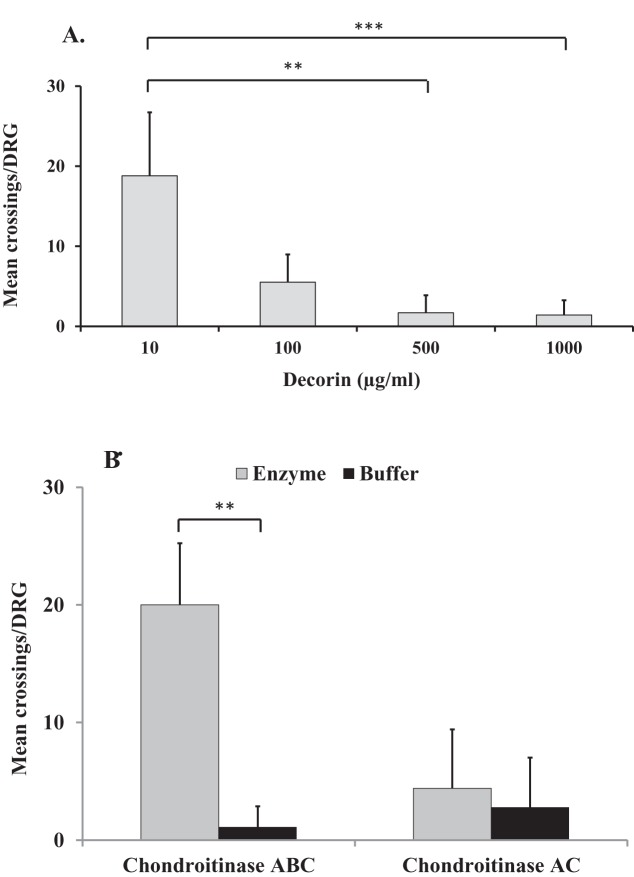
The effect of glycosylated and non-glycosylated decorin on DRG neurite growth. Graphs showing the mean ± S.D. of number of neurite crossings per DRG when cultured on (**A**) ‘decorin’ at a range of concentrations (10–1000 µg/ml) without any enzymatic treatment; (**B**) ‘decorin’ at 500 µg/ml following chondroitinase ABC and chondroitinase AC treatment (both at 250 mU/ml) or in buffer; **P<0.005, ***P<0.0001.

Substrate choice experiments showed that ‘decorin’ inhibited DRG neurite growth in a dose-dependent manner (Figure 1A). Significant decreases in the average number of neurite crossings per DRG were observed between ‘decorin’ concentrations of 10 and 500 µg/ml (*P*=0.0012) and 10 and 1000 µg/ml (*P*<0.0001). No significant difference on DRG neurite growth was observed between ‘decorin’ concentrations of 500 and 1000 µg/ml.

Since maximum inhibition of DRG neurite growth was observed using ‘decorin’ at 500 µg/ml, experiments were repeated using this concentration to determine the effect of enzymatic removal of GAG chains from the ‘decorin’ on neurite growth. Figure 1(B) shows that chondroitinase ABC (which cleaves both CS and DS GAGs) at 250 mU/ml significantly reduced the inhibitory effect of ‘decorin’ on DRG neurite growth compared with no enzymatic treatment (*P*=0.0013). In contrast, treatment with chondroitinase AC (which cleaves CS GAGs only) did not reverse the inhibitory effects of ‘decorin’ on DRG neurite growth (Figure 1B).

**Figure 2:**
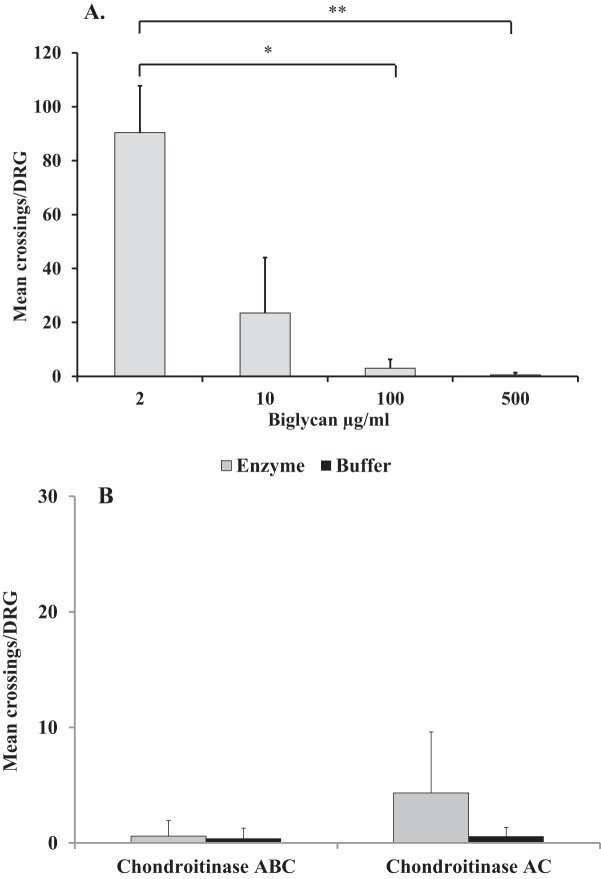
The effect of glycosylated and non-glycosylated biglycan on DRG neurite growth. Graphs showing the mean ± S.D. of number of neurite crossings per DRG when cultured on **(A**) ‘biglycan’ at a range of concentrations (2–500 µg/ml) without any enzymatic treatment; **(B)** ‘biglycan’ at 500 µg/ml following either chondroitinase ABC or chondroitinase AC digestion (both at 250 mU/ml) or in buffer; *P<0.05, **P<0.005.

The experiments were repeated using ‘biglycan’ instead of ‘decorin’ as the test substrate and a similar dose-dependent pattern of DRG neurite growth inhibition was observed (Figure 2A). That is, there was a significant decrease in neurite crossings when the ‘biglycan’ concentration was increased from 2 to 100 µg/ml (*P*=0.0114) and when it was increased from 2 to 500 µg/ml (*P*=0.0007). No significant difference on DRG neurite growth was observed between ‘biglycan’ concentrations of 100 and 500 µg/ml. Again, the effect on DRG neurite growth of the CS and DS GAG chain components of the ‘biglycan’ preparation were assessed by using ‘biglycan’ at 500 µg/ml (as previously used to assess ‘decorin’) followed by either chondroitinase ABC or chondroitinase AC (both at 250 mU/ml). As can be seen in Figure 2(B), inhibition of DRG neurite growth was not reversed following removal of CS or CS and DS GAG chains and the inhibitory effect of the ‘biglycan’ molecule was maintained.

### Western blot analysis of the glycosaminoglycan chains of the commercially sourced ‘decorin’ and ‘biglycan’

**Figure 3:**
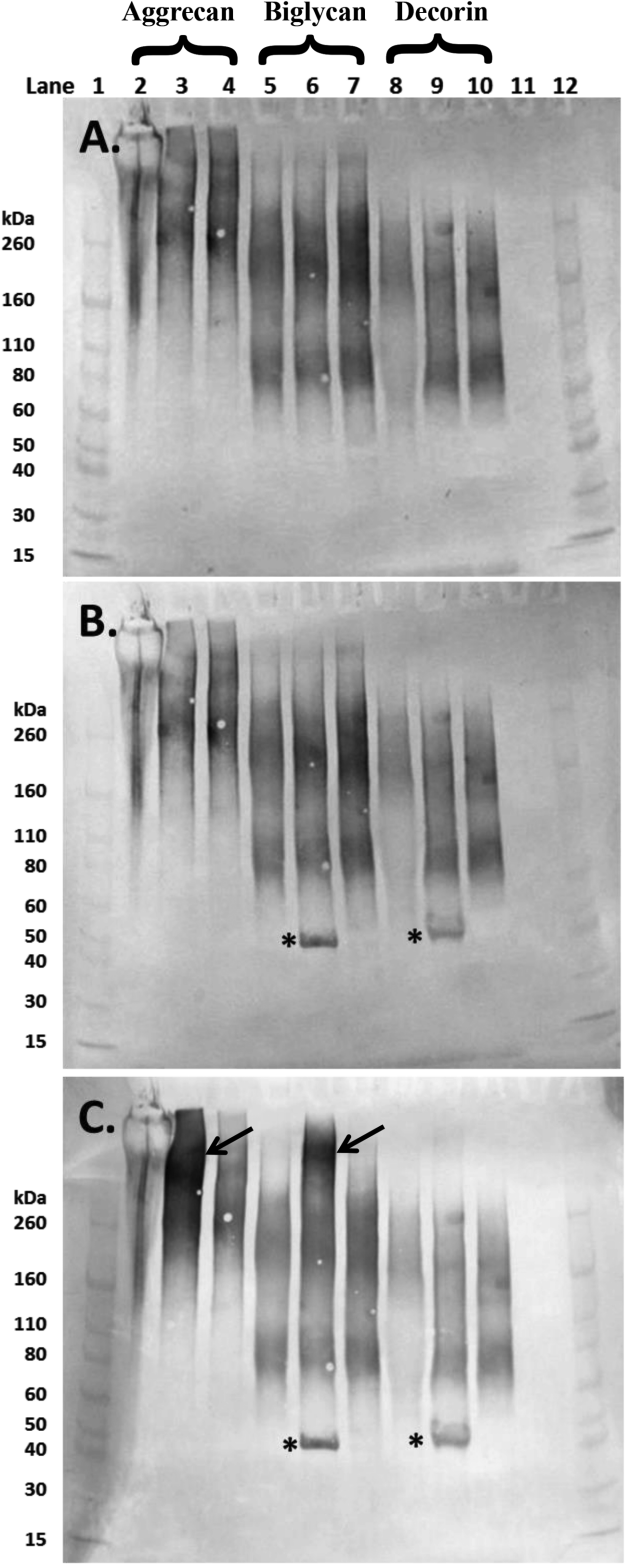
Western blot images of aliquots of purified aggrecan isolated from a human intervertebral disc (lanes 2–4), and the commercially sourced ‘biglycan’ (lanes 5–7) and ‘decorin’ (lanes 8–10) following separation on 4–12% Bis-Tris gel PGs in lanes 2, 5 and 8 were undigested whereas those in lanes 3, 6 and 9 and 4, 7 and 10 had been digested with chondroitinase ABC and chondroitinase AC respectively (both 250 mU/ml). Membranes were probed sequentially with monoclonal antibodies to (**A**) over-sulfated KS heptasaccharides (5D4); (**B**) 4-sulfated unsaturated disaccharide CS neo-epitopes (2B6) and **(C**) 6-sulfated unsaturated disaccharide CS neo-epitopes (3B3). Lanes 1 and 12 contain molecular weight standards. The expected molecular weight of the intact core protein of both biglycan and decorin is approximately 45 kDa (*). Aggrecan bands were particularly prominent in lane 3 (4C) following chondrointase ABC treatment (arrow), similar bands were seen in the ‘biglycan’ preparation (lane 6, arrow).

Western blot analysis of the ‘decorin’, ‘biglycan’ and aggrecan revealed the presence of KS, in all three PGs with clear 5D4-positivity (Figure 3A). Although the presence of KS was expected within the aggrecan preparation, it was not in the two SLRP preparations as decorin is reported to contain only one GAG chain of CS/DS and biglycan two chains of CS/DS [40]. The bands for samples that had undergone enzymatic pre-treatment with either chondroitinase ABC or AC appeared to be more intense (Figure 3A; lanes 3 and 4, 6 and 7 and 9 and 10) indicating that removal of the CS and DS GAG chains exposed the KS containing sugars further. The bands associated with aggrecan ran from above the 260 kDa molecular marker down to approximately 140 kDa. The KS-positive PGs in ‘biglycan’ and ‘decorin’ ran from just above the 260 kDa standard, down to approximately 60 kDa (Figure 3A).

When the membrane was probed for 4-sulfated CS using the antibody 2B6 (after chondroitinase ABC pre-treatment), additional bands were seen at approximately 45 kDa in both ‘biglycan’ and ‘decorin’ (Figure 3B). This is the expected molecular weight of the core protein of both decorin and biglycan. Since these bands were not visible in the preparations treated with chondroitinase AC, the DS GAG chains must have been more common than CS on the ‘biglycan’ and ‘decorin’ components of these commercial SLRP preparations. The membranes were also probed for the 6-sulfated CS with the antibody 3B3 and following chondroitinase ABC digestion where both aggrecan and ‘biglycan’ had increased immunoreactivity relating to these 6-sulfated neo-epitopes at high molecular weights (>260 kDa) whereas as expected ‘decorin’ did not (Figure 3C).

### MS characterization of commercially sourced ‘decorin’ and ‘biglycan’

**Figure 4:**
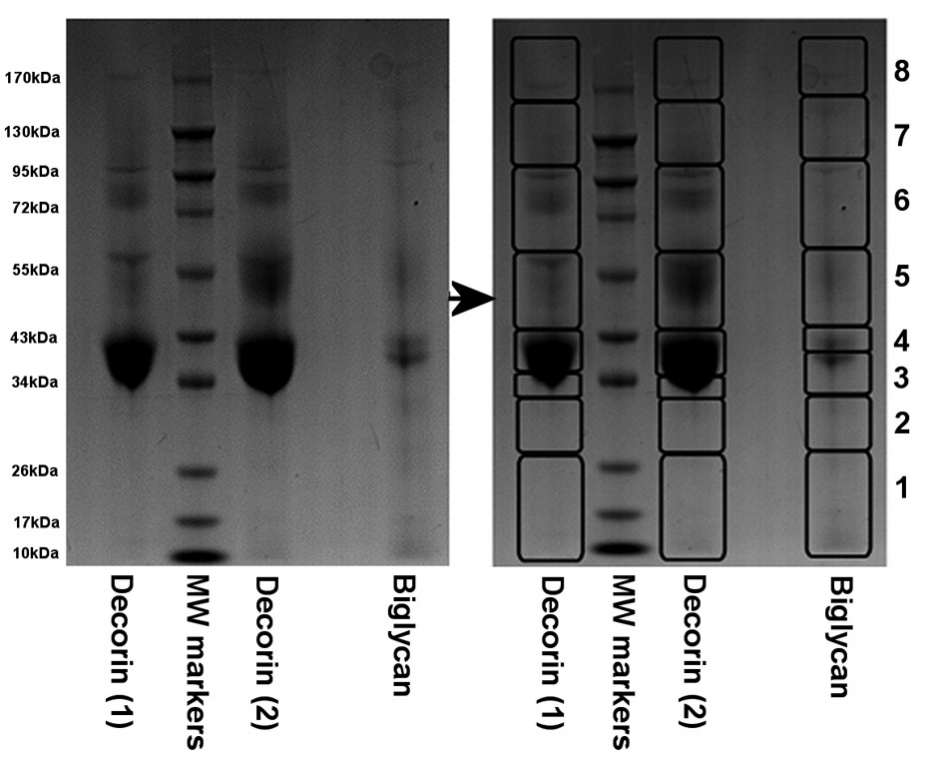
Image of one of the 12.5% SDS-PAGE stained with Coomassie Blue following electrophoretic separation of the commercially sourced ‘decorin’ (1st batches (1) and 2nd batch (2)) and ‘biglycan’ after enzymatic digestion with chondroitinase ABC, keratanase and keratanase II (removing all CS, DS and KS GAG chains) Protein slices as outlined (1–8) were dissected from each lane and then prepared for MS analyses.

Mass spectrometric analysis of gel slices excised from sample lanes containing the two batches of ‘decorin’ preparations (Figure 4) resulted in the identification of several proteins with statistical significance (summarized in Table 2 and in full in Supplementary Table S1). Biglycan was the most easily detectable protein in the majority of the ‘decorin’ gel slices and fibromodulin and aggrecan were also detectable in several slices from each of the two preparations (Supplementary Tables S1a and S1c). Surprisingly, decorin itself was only detected with significance in one out of eight gel slices from ‘decorin’ (2nd batch), whereas the decorin peptides detected in the ‘decorin’ (1st batch) preparation did not meet the criteria required for a statistically significant protein identification (see “slice 4”, Supplementary Table S1b). Although it is not possible to derive accurate measures of relative protein quantity using this approach, it seems fair to assume that decorin is a minor component of both commercial batches of ‘decorin’ preparations, since it was detected with far fewer peptides than the other proteins that were detected in each sample.

**Table 2:** A summary of the proteins that were identified with significance in ‘biglycan’ and ‘decorin’ preparations by MS analysis Only proteins that were identified with statistical significance (i.e. *P*<0.05) are shown. The number of MS peptides refers to the number of peptides that were matched to each protein of bovine origin and the number of MS/MS peptides refers to the number of peptides for which it was possible to generate matching ions.

Sample	Proteins detected with significance^*^	Number of MS peptides	Number of MS/MS peptides
‘Decorin’ (1st batch)	Biglycan	14	6
	Fibromodulin	8	4
	Aggrecan	14	4
‘Decorin’ (2nd batch)	Biglycan	15	8
	Fibromodulin	9	5
	Aggrecan	18	5
	Decorin	5	2
‘Biglycan’	Biglycan	16	7
	Fibromodulin	8	4
	Aggrecan	23	8
	Decorin	9	3

*Due to inherent issues with database redundancy, significant protein matches are often made to multiple species (because of sequence homology). In this instance, because we know the preparations were of bovine origin, only significant hits that were assigned to this species are summarized here. For full protein and peptide summaries of each gel slice, please refer to Supplementary Table S1.

**Figure 5:**
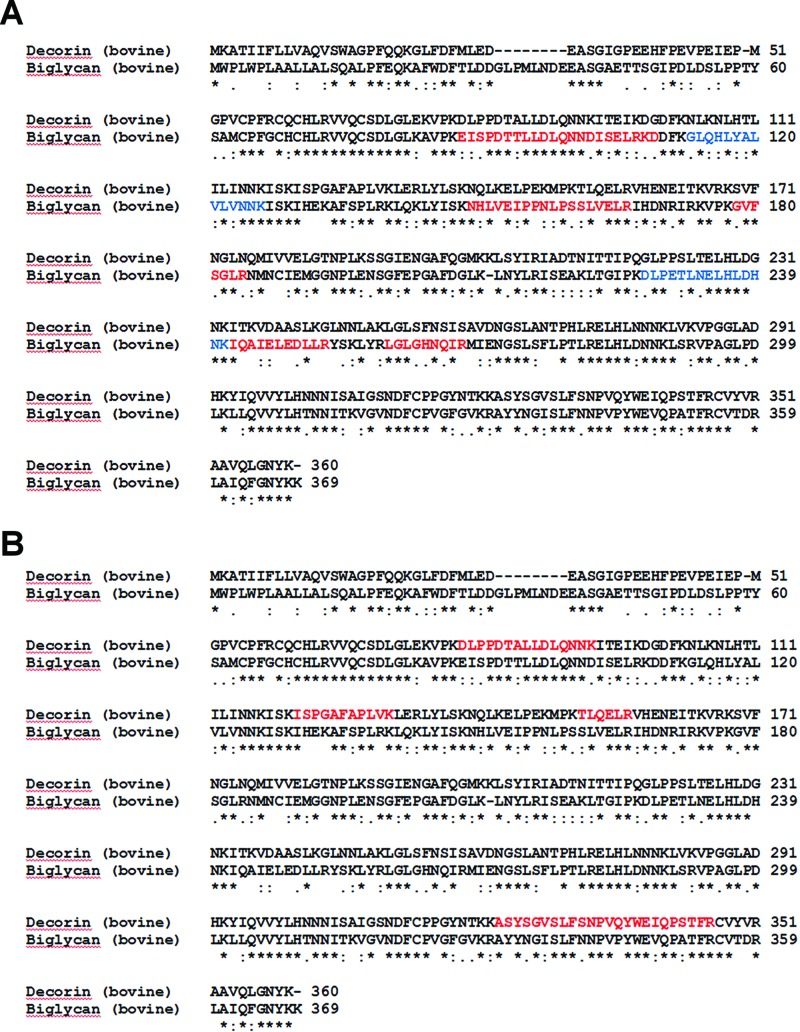
Sequence alignments highlighting biglycan peptides identified by MS analysis The ClustalW2 sequence alignment tool was used to align the amino acid sequence for bovine decorin (P21793) and bovine biglycan (P21809). **(A**) Biglycan peptides that were identified in both ‘decorin’ samples are shown in red. Additional peptides identified only in the ‘decorin’ (2nd batch) preparation are shown in blue. (**B**) Decorin peptides that were identified in the ‘biglycan’ sample are shown in red.

Similar to the ‘decorin’ (2nd batch) preparation, MS analysis of the gel slices excised from the ‘biglycan’ sample lane resulted in the identification of significant peptides matching to biglycan, fibromodulin, aggrecan and decorin (Table 2 and Supplementary Table S1). Though some degree of amino sequence similarity (69%) and identity (52%) exists between bovine biglycan and decorin, an alignment of the two sequences confirmed that the peptides identified in each case were not common to both proteins (Figure 5).

### Validation by Western blot of the MS data for the commercially sourced ‘decorin’ and ‘biglycan’

**Figure 6:**
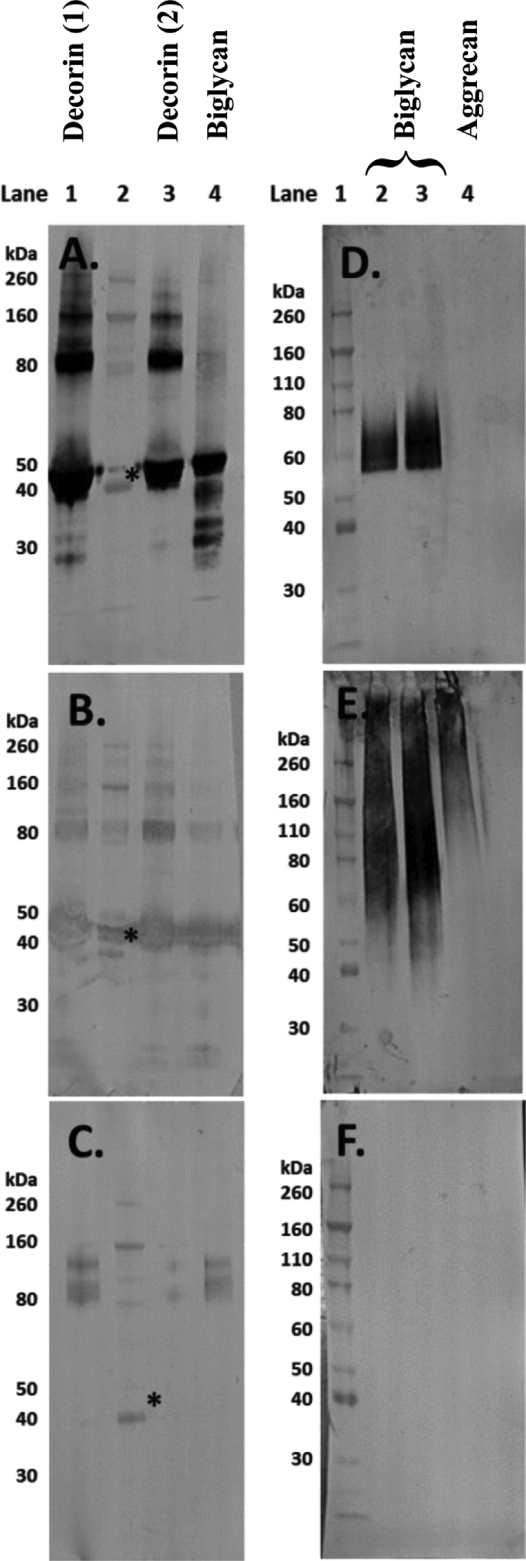
Western blot images of aliquots of the commercially sourced ‘decorin’ and ‘biglycan’ and purified human aggrecan following separation on 4–12% Bis-Tris gels The PGs in (**A**)–(**C**) had been digested with chondroitinase ABC, keratanase and keratanase II (removing CS, DS and KS GAG chains). Lanes 1 and 3 of (A)–(C) contain different batches of ‘decorin’, lane 4 contains ‘biglycan’ and lane 2 contains the molecular weight standards. The expected molecular weight of the intact core protein of both biglycan and decorin is approximately 45 kDa (*). The membranes were probed with antibodies to (A) biglycan (PR-85); (B) decorin (DS-1) and (**C**) aggrecan (6B4). For the membranes in (**D**)–(**F**), lane 1 contains the molecular weight standards; lanes 2 and 3 contain ‘biglycan’ without and with prior enzymatic digestion with chondroitinase ABC respectively and in lane 4 is purified human aggrecan without prior digestion. These membranes were probed with **(**D) fibromodulin; (E) 1B4, which detects less-sulfated KS and (F) lumican.

Subsequent to the unexpected MS findings, further Western blot analyses were carried out on the PG preparations. Membranes were probed for biglycan and decorin and although ‘biglycan’ contained biglycan and ‘decorin’ contained decorin, the presence of biglycan in ‘decorin’ and decorin in ‘biglycan’ (Figure 6A and B respectively) was confirmed and validated the findings of the mass spectrometric analysis (Table 2). Although the most intense immunoreactivity for the ‘decorin’ and ‘biglycan’ protein cores was observed at the expected molecular weight of approximately 45 kDa, higher molecular weight bands were also detectable for these proteins (Figure 6A and B). One of the other main findings from MS was the presence of aggrecan within both SLRP preparations, and by probing with 6B4 (a monoclonal antibody to the amino acid sequence 413–424 of human aggrecan) its presence was also confirmed by Western blot (Figure 6C).

Further Western blot analysis of ‘biglycan’, both with and without chondroitinase ABC pre-treatment and undigested purified aggrecan as a positive control, clearly showed the presence of fibromodulin in ‘biglycan’ but none in the aggrecan preparation (Figure 6D). Additionally, the presence of low-sulfated KS (as shown with the 1B4 antibody) was detected in both ‘biglycan’ and aggrecan with its presence being detected at molecular weights as low as 40–50 kDa in ‘biglycan’ while in aggrecan, as expected, it was only detected at molecular weights of 100 kDa and greater. As a final validation step, membranes were probed for lumican; an SLRP that was not detected via MS in ‘biglycan’ and that should not be present in the purified aggrecan preparation. No positive immunoreactivity for lumican in either ‘biglycan’ or aggrecan was detected (Figure 6F).

## Discussion

Molecules that contain GAG chains have previously been shown to influence neuronal growth with those containing CS in particular being implicated in neurite inhibition [8]. The aim of the present study was to establish the effect on neurite growth of the two Class 1 SLRP family members, the CS/DS-containing SLRPs, decorin and biglycan. Initial findings did corroborate with those previously established with aggrecan [3,31], in that both commercially sourced ‘decorin’ and ‘biglycan’, without any enzymatic treatment, inhibited DRG neurite growth. To ensure that the observed inhibition was due to the presence of these CS/DS GAG chains, ‘decorin’ and ‘biglycan’ were enzymatically deglycosylated. For decorin, this resulted, as expected, in a reduction in its inhibitory effect on neurite growth. In contrast, this effect was not observed with ‘biglycan’. This triggered further investigations we have described, using analytical techniques to decipher this anomaly.

Western blot analysis of ‘decorin’, ‘biglycan’ and aggrecan with varying degrees of glycosylation revealed the presence of KS chains on all three PG preparations. Although KS was expected to be present within the aggrecan molecule, it should not have been present in either of the SLRP preparations. In all three PG analyses, the Western blot bands appeared diffuse (Figure 3A), reflecting a variety of molecular weight moieties containing KS. Following digestion with chondroitinase ABC or AC, the intensity of the Western blot bands increased indicating that CS and DS GAGs had been present with their removal exposing additional KS epitopes that reacted with the KS-specific 5D4 antibody. Biglycan and decorin core proteins (at a molecular weight of approximately 45 kDa) were only visible on the membranes following chondroitinase ABC digestion (Figure 3B), implying that removal of DS GAG chains (but not CS chains) exposed these core proteins. Interestingly, following chondroitinase ABC digestion, the ‘biglycan’ preparation appeared to have increased immunoreactivity for the 6-sulfated 3B3 neo-epitope (Figure 3C, arrows) at high molecular weights similarly to that seen with aggrecan. From these results, it appears that the ‘biglycan’ may contain a higher proportion of high molecular weight molecules, such as aggrecan, with GAG chains containing more C-6-S than the ‘decorin’ preparation. This, in addition to the greater amounts of high molecular weight KS containing PGs in ‘biglycan’, may explain the difference observed between ‘biglycan’ and ‘decorin’ when treated enzymatically to overcome neurite inhibition. These findings may also provide an explanation for the lower than expected presence of uronic acid in ‘decorin’ (20–27%) and even lower presence in ‘biglycan’ (14%), as detailed in their relevant product specifications. Typically, the CS and DS GAG chains of decorin and biglycan would contain 50% uronic acid [41].

The combination of MS and Western blotting used in the present study has demonstrated that commercial preparations of ‘biglycan’ and ‘decorin’ bought and used in studies in our laboratory actually contained many contaminating substances, at least in these specific batches. The molecules we concentrated on validating were other PG family members identified via MS. Hence, the ‘decorin’ also contained biglycan, fibromodulin and aggrecan, while the ‘biglycan’ also contained fibromodulin, aggrecan and decorin, at least. This renders these preparations useless for the original purpose of our study which was to unravel the contributions that different members of the PG family, known to occur in the human intervertebral disc, may make to controlling neurite extension and nerve growth.

The impact of our findings may be huge, not in the manner we originally intended but, rather in the amount of time, money and samples that have been wasted. Most importantly, however, it may affect the possibility of erroneous information entering the scientific arena. Concern about the purity and quality of commercial chemicals and biochemicals has previously been expressed by other researchers. For example, D’Hondt et al. [42] highlighted the impact that contaminants may have in peptide medicines, not only with respect to their presence within the approved and clinically used products, but also during the initial drug discovery phase. During this initial phase, the presence of contaminants may greatly influence functionality studies thus resulting in conclusions that are incorrect. We know of publications that draw important mechanistic conclusions on the influence of decorin on the control of sensory neuron growth, wound-healing in glaucoma, prevention of the development of vitreoretinopathy and susceptibility to malaria. All appeared to use the same source of ‘decorin’ as we did. If their decorin was as impure as ours, are their results still valid? Do scientists have to check the purity of every reagent? Some researchers do prepare and validate the purity of their own preparations [43]. Unfortunately most research scientists do not have the breadth of experience, knowledge or facilities to undertake this and so obtain such materials as we did, from a commercial source in good faith.

There are many sophisticated analytical techniques in the scientific world and it is hoped that companies will use these to provide more accurate details of the purity and constituents of their reagents and chemicals. Inherently there will be a cost to the consumer, but on balance, the reduction in time and funds spent on investigating the effects of ‘impure’ molecules within the scientific community, possibly resulting in inaccurate conclusions and ‘flawed’ studies, will offset this cost. Without this, the effect of contaminants within reagents may alter or confound the observed effect of the molecule being investigated; potentially leading to erroneous interpretation of data such as in the elucidation of mechanisms of action or the contribution a particular molecule may play in key biological processes.

## Abbreviations

CS: chondroitin sulfate
DRG: dorsal root ganglion
DS: dermatan sulfate
ECM: extracellular matrix
GAG: glycosaminoglycan
GuHCl: guanidine hydrochloride
KS: keratan sulfate
PG: proteoglycan
SLRP: small leucine-rich proteoglycan

## References

[1] Carulli D., Laabs T., Geller H.M. and Fawcett J.W. (2005) Chondroitin sulfate proteoglycans in neural development and regeneration. Curr. Opin. Neurobiol. 15, 116–120 15721753 10.1016/j.conb.2005.01.014

[2] Kubota Y., Morita T., Kusakabe M., Sakakura T. and Ito K. (1999) Spatial and temporal changes in chondroitin sulfate distribution in the sclerotome play an essential role in the formation of migration patterns of mouse neural crest cells. Dev. Dyn. 214, 55–65 9915576 10.1002/(SICI)1097-0177(199901)214:1<55::AID-DVDY6>3.0.CO;2-E

[3] Johnson W.E., Caterson B., Eisenstein S.M., Hynds D.L., Snow D.M. and Roberts S. (2002) Human intervertebral disc aggrecan inhibits nerve growth in vitro. Arthritis Rheum. 46, 2658–2664 12384924 10.1002/art.10585

[4] Melrose J., Roberts S., Smith S., Menage J. and Ghosh P. (2002) Increased nerve and blood vessel ingrowth associated with proteoglycan depletion in an ovine anular lesion model of experimental disc degeneration. Spine (Phila Pa 1976) 27, 1278–1285 12065974 10.1097/00007632-200206150-00007

[5] Stefanakis M., Al Abbasi M., Harding I., Pollintine P., Dolan P., Tarlton J. (2012) Annulus fissures are mechanically and chemically conducive to the ingrowth of nerves and blood vessels. Spine (Phila Pa 1976) 37, 1883–1891 22706090 10.1097/BRS.0b013e318263ba59

[6] Lemons M.L., Barua S., Abanto M.L., Halfter W. and Condic M.L. (2005) Adaptation of sensory neurons to hyalectin and decorin proteoglycans. J. Neurosci. 25, 4964–4973 15901777 10.1523/JNEUROSCI.0773-05.2005 PMC6724852

[7] Davies J.E., Tang X., Denning J.W., Archibald S.J. and Davies S.J. (2004) Decorin suppresses neurocan, brevican, phosphacan and NG2 expression and promotes axon growth across adult rat spinal cord injuries. Eur. J. Neurosci. 19, 1226–1242 15016081 10.1111/j.1460-9568.2004.03184.x

[8] Coles C.H., Shen Y., Tenney A.P., Siebold C., Sutton G.C., Lu W. (2011) Proteoglycan-specific molecular switch for RPTPσ clustering and neuronal extension. Science 332, 484–488 21454754 10.1126/science.1200840 PMC3154093

[9] Cheung K.M., Karppinen J., Chan D., Ho D.W., Song Y.Q., Sham P. (2009) Prevalence and pattern of lumbar magnetic resonance imaging changes in a population study of one thousand forty-three individuals. Spine (Phila Pa 1976) 34, 934–940 19532001 10.1097/BRS.0b013e3181a01b3f

[10] Vos T., Flaxman A.D., Naghari M., Lozano R., Michaud C., Ezzati M. (2012) Years lived with disability (YLDs) for 1160 sequelae of 289 diseases and injuries 1990-2010: a systematic analysis for the Global Burden of Disease Study 2010. Lancet 380, 2163–2196 23245607 10.1016/S0140-6736(12)61729-2 PMC6350784

[11] Hong J., Reed C., Novick D. and Happich M. (2013) Costs associated with treatment of chronic low back pain: an analysis of the UK General Practice Research Database. Spine (Phila Pa 1976) 38, 75–82 23038621 10.1097/BRS.0b013e318276450f

[12] Maniadakis N. and Gray A. (2000) The economic burden of back pain in the UK. Pain 84, 95–103 10601677 10.1016/S0304-3959(99)00187-6

[13] Livshits G., Popham M., Malkin I., Sambrook P.N., MacGregor A.J., Spector T. (2011) Lumbar disc degeneration and genetic factors are the main risk factors for low back pain in women: the UK Twin Spine Study. Ann. Rheum. Dis. 70, 1740–1745 21646416 10.1136/ard.2010.137836 PMC3171106

[14] Adams M.A. (1994) Modern manual therapy: biomechanics of the lumbar motion segment, 2nd edn, pp. 109–129, Churchill Livingstone, Edinburgh

[15] Urban J. and Roberts S. (2003) Cells of the intervertebral disc: making the best of a bad environment. Biochemist 25, 15–17

[16] Ashton I.K., Roberts S., Jaffray D.C., Polak J.M. and Eisenstein S.M. (1994) Neuropeptides in the human intervertebral disc. J. Orthop. Res. 12, 186–192 8164090 10.1002/jor.1100120206

[17] Roberts S., Evans H., Trivedi J. and Menage J. (2006) Histology and pathology of the human intervertebral disc. J. Bone Joint Surg. Am. 88, 10–14 10.2106/JBJS.F.00019 16595436

[18] Freemont A.J., Peacock T.E., Goupille P., Hoyland J.A., O’Brien J. and Jayson M.I. (1997) Nerve ingrowth into diseased intervertebral disc in chronic back pain. Lancet 350, 178–181 9250186 10.1016/s0140-6736(97)02135-1

[19] Lama P., Le Maitre C.L., Dolan P., Tarlton J.F., Harding I.J. and Adams M.A. (2013) Do intervertebral discs degenerate before they herniate, or after? Bone Joint J. 95-B, 1127–1133 23908431 10.1302/0301-620X.95B8.31660

[20] Roberts S., Beard H.K. and O’Brien J.P. (1982) Biochemical changes of intervertebral discs in patients with spondylolisthesis or with tears of the posterior annulus fibrosus. Ann. Rheum. Dis. 41, 78–85 7065733 10.1136/ard.41.1.78 PMC1000869

[21] Donohue P.J., Jahnke M.R., Blaha J.D. and Caterson B. (1988) Characterization of link protein(s) from human intervertebral-disc tissues. Biochem. J. 251, 739–747 3415643 10.1042/bj2510739 PMC1149066

[22] Roberts S. (1990) Methods in cartilage research: sampling of the intervertebral disc, pp. 17–19, Academic Press Ltd, London

[23] Brown S., Melrose J., Caterson B., Roughley P., Eisenstein S.M. and Roberts S. (2012) A comparative evaluation of the small leucine-rich proteoglycans of pathological human intervertebral discs. Eur. Spine J. 21, S154–S159 22358337 10.1007/s00586-012-2179-1 PMC3326086

[24] Singh K., Masuda K., Thonar E.J.-M.A., An H.S. and Cs-Szabo G. (2009) Age-related changes in the extracellular matrix of nucleus pulposus and anulus fibrosus of human intervertebral disc. Spine (Phila Pa 1976) 34, 10–16 19127156 10.1097/BRS.0b013e31818e5ddd PMC2837353

[25] Sztrolovics R., Alini M., Mort J.S. and Roughley P.J. (1999) Age-related changes in fibromodulin and lumican in human intervertebral discs. Spine (Phila Pa 1976) 24, 1765–1771 10488504 10.1097/00007632-199909010-00003

[26] Melrose J., Smith S.M., Fuller E.S., Young A.A., Roughley P.J., Dart A. (2007) Biglycan and fibromodulin fragmentation correlates with temporal and spatial annular remodelling in experimentally injured ovine intervertebral discs. Eur. Spine J. 16, 2193–2205 17899219 10.1007/s00586-007-0497-5 PMC2140141

[27] Erwin W.M., DeSouza L., Funabashi M., Kawchuk G., Karim M.Z., Kim S. (2015) The biological basis of degenerative disc disease: proteomic and biomechanical analysis of the canine intervertebral disc. Arthritis Res. Ther. 17, 240 26341258 10.1186/s13075-015-0733-z PMC4560915

[28] Esmaeili M., Berry M., Logan A. and Ahmed Z. (2014) Decorin treatment of spinal cord injury. Neural Regener. Res. 9, 1653–1656 10.4103/1673-5374.141797 PMC4211183 25374584

[29] Amenta A.R., Creely H.E., Mercado M.L., Hagiwara H., McKechnie B.A., Lechner B.E. (2012) Biglycan is an extracellular MuSK binding protein important for synapse stability. J. Neurosci. 32, 2324–2334 22396407 10.1523/JNEUROSCI.4610-11.2012 PMC3313673

[30] Kritis A., Kapoukranidou D., Michailidou B., Hatzisotiriou A. and Albani M. (2010) Sciatic nerve crush evokes a biphasic TGF-beta and decorin modulation in the rat spinal cord. Hippokratia 14, 37–41 20411058 PMC2843569

[31] Johnson W.E.B., Sivan S., Wright K.T., Eisenstein S.M., Maroudas A. and Roberts S. (2006) Human intervertebral disc cells promote nerve growth over substrata of human intervertebral disc aggrecan. Spine (Phila Pa 1976) 31, 1187–1193 16688030 10.1097/01.brs.0000217669.04903.61

[32] Wright K.T., Uchida K., Bara J.J., Roberts S., El Masri W. and Johnson W.E.B. (2014) Spinal motor neurite outgrowth over glial scar inhibitors is enhanced by coculture with bone marrow stromal cells. Spine J. 14, 1722–1733 24462452 10.1016/j.spinee.2014.01.021

[33] Lowry O.H., Rosebrough N.J., Farr A.L. and Randall R.J. (1951) Protein measurement with the Folin phenol reagent. J. Biol. Chem. 193, 265–275 14907713

[34] Jahnke M.R. and McDevitt C.A. (1988) Proteoglycans of the human intervertebral disc. Electrophoretic heterogeneity of the aggregating proteoglycans of the nucleus pulposus. Biochem. J. 251, 347–356 3041961 10.1042/bj2510347 PMC1149009

[35] Sivan S.S., Tsitron E., Wachtel E., Roughley P.J., Sakkee N., van der Ham F. (2006) Aggrecan turnover in human intervertebral disc as determined by the racemization of aspartic acid. J. Biol. Chem. 281, 13009–13014 16537531 10.1074/jbc.M600296200

[36] Bayliss M.T. and Ali S.Y. (1978) Isolation of proteoglycans from human articular cartilage. Biochem. J. 169, 123–132 24438 10.1042/bj1690123 PMC1184201

[37] Maroudas A., Bayliss M.T., Uchitel-Kaushansky N., Schneiderman R. and Gilav E. (1998) Aggrecan turnover in human articular cartilage: use of aspartic acid racemization as a marker of molecular age. Arch. Biochem. Biophys. 350, 61–71 9466821 10.1006/abbi.1997.0492

[38] Melrose J., Fuller E.S., Roughley P.J., Smith M.M., Kerr B., Hughes C.E. (2008) Fragmentation of decorin, biglycan, lumican and keratocan is elevated in degenerate human meniscus, knee and hip articular cartilages compared with age-matched macroscopically normal and control tissues. Arthritis Res. Ther. 10, R79 18620607 10.1186/ar2453 PMC2575625

[39] Shevchenko A., Wilm M., Vorm O. and Mann M. (1996) Mass spectrometric sequencing of proteins silver-stained polyacrylamide gels. Anal. Chem. 68, 850–858 8779443 10.1021/ac950914h

[40] Iozzo R. V. (1999) The biology of the small leucine-rich proteoglycans. Functional network of interactive proteins. J. Biol. Chem. 274, 18843–18846 10383378 10.1074/jbc.274.27.18843

[41] Caterson B. (2012) Fell-Muir Lecture: chondroitin sulphate glycosaminoglycans: fun for some and confusion for others. Int. J. Exp. Pathol. 93, 1–10 22264297 10.1111/j.1365-2613.2011.00807.x PMC3311016

[42] D’Hondt M., Bracke N., Taevernier L., Gevaert B., Verbeke F., Wynendaele E. (2014) Related impurities in peptide medicines. J. Pharm. Biomed. Anal. 101, 2–30 25044089 10.1016/j.jpba.2014.06.012

[43] Schaefer L., Babelova A., Kiss E., Hausser H.J., Baliova M., Krzyzankova M. (2005) The matrix component biglycan is proinflammatory and signals through toll-like receptors 4 and 2 in macrophages. J. Clin. Invest. 115, 2223–2233 16025156 10.1172/JCI23755 PMC1174916

[44] Poole A.R., Webber C., Pidoux I., Choi H. and Rosenberg L.C. (1986) Localization of a dermatan sulfate proteoglycan (DS-PGII) in cartilage and the presence of an immunologically related species in other tissues. J. Histochem. Cytochem. 34, 619–625 3701029 10.1177/34.5.3701029

[45] Young A.A., Smith M.M., Smith S.M., Cake M.A., Ghosh P., Read R.A. (2005) Regional assessment of articular cartilage gene expression and small proteoglycan metabolism in an animal model of osteoarthritis. Arthritis Res. Ther. 7, R852–R861 15987487 10.1186/ar1756 PMC1175037

[46] Little C.B., Hughes C.E., Curtis C.L., Janusz M.J., Bohne R. and Wang-Weigand S. (2002) Matrix metalloproteinases are involved in C-terminal and interglobular domain processing of cartilage aggrecan in late stage cartilage degradation. Matrix Biol. 21, 271–288 12009333 10.1016/s0945-053x(02)00004-5

[47] Sztrolovics R., White R.J., Poole A.R., Mort J.S. and Roughley P.J. (1999) Resistance of small leucine-rich repeat proteoglycans to proteolytic degradation during interleukin-1-stimulated cartilage catabolism. Biochem. J. 339, 571–577 10215595 PMC1220192

[48] Caterson B., Christner J.E. and Baker J.R. (1983) Identification of a monoclonal antibody that specifically recognizes corneal and skeletal keratan sulphate. Monoclonal antibodies to cartilage proteoglycan. J. Biol. Chem. 258, 8848–8854 6223038

[49] Young R.D., Gealy E.C., Liles M., Caterson B., Ralphs J.R. and Quantock A.J. (2007) Keratan sulfate glycosaminoglycan and the association with collagen fibrils in rudimentary lamellae in the developing avian cornea. Invest. Ophthalmol. Vis. Sci. 48, 3083–3088 17591877 10.1167/iovs.06-1323

[50] Caterson B., Christner J.E., Baker J.R. and Couchman J.R. (1985) Production and characterization of monoclonal antibodies directed against connective tissue proteoglycans. Fed. Proc. 44, 386–393 2578417

[51] Ilic M.Z., Handley C.J., Robinson H.C. and Mok M.T. (1992) Mechanism of catabolism of aggrecan by articular cartilage. Arch. Biochem. Biophys. 294, 115–122 1550337 10.1016/0003-9861(92)90144-l

